# Silencing of S100A4, a metastasis-associated protein, in endothelial cells inhibits tumor angiogenesis and growth

**DOI:** 10.1007/s10456-013-9372-7

**Published:** 2013-08-09

**Authors:** Takahiro Ochiya, Keizo Takenaga, Hideya Endo

**Affiliations:** 1 Division of Molecular and Cellular Medicine, National Cancer Center Research Institute, 5-1-1, Tsukiji, Chuo-ku, Tokyo 104-0045 Japan; 2 Department of Life Science, Faculty of Medicine, Shimane University, 89-1 Enya, Izumo, 693-8501 Japan; 3 Institute of Medical Science, University of Tokyo, 4-6-1 Shirokanedai, Minato-ku, Tokyo 108-8639 Japan

**Keywords:** Angiogenesis, S100A4, siRNA, Endothelial cells, Tumor, Therapy

## Abstract

Endothelial cells express S100A4, a metastasis-associated protein, but its role in angiogenesis remains to be elucidated. Here we show that knockdown of S100A4 in mouse endothelial MSS31 cells by murine specific small interference RNA (mS100A4 siRNA) markedly suppressed capillary-like tube formation in vitro, in early stage after the treatment, along with down- and up-regulation of some of the pro-angiogenic and anti-angiogenic gene expression, respectively. Of particular note is that intra-tumor administration of the mS100A4 siRNA in a human prostate cancer xenograft significantly reduced tumor vascularity and resulted in the inhibition of tumor growth. These findings show that S100A4 in endothelial cells is involved in tube formation, and suggest its potential as a molecular target for inhibiting tumor angiogenesis, which warrants further development of endothelial S100A4-based strategies for cancer treatment.

**Electronic supplementary material:**

The online version of this article (doi:10.1007/s10456-013-9372-7) contains supplementary material, which is available to authorized users.

## Introduction

S100A4, a member of the S100 family of EF-hand calcium-binding proteins, is involved in the regulation of a variety of biological processes, including tumor progression and metastasis [1]. It is highly expressed in various metastatic tumor cells and its elevated expression is associated with the poor prognosis of different types of human cancers [2–7]. The involvement of S100A4 in the promotion of tumor metastasis has been demonstrated by several approaches [8–11]. Furthermore, recent studies on mice that lack the S100A4 gene [12], and on mice carrying tumor cells subjected to siRNA-mediated S100A4-downregulation [13–18], have shown suppression of tumor development and metastasis formation. Although the precise mechanism by which S100A4 stimulates invasion and metastasis remains unknown, an extracellular role for S100A4 suggested by studies showing the secretion of S100A4 by cancer cells and the presence of S100A4 in the serum of cancer patients is intriguing [19]. In fact, the exogenous addition of S100A4 stimulates endothelial cell motility in vitro [20] and induces corneal neovascularization [19] and metastasis formation in vivo [21]. On the other hand, little is known about the effect of endothelial S100A4 on angiogenesis. We previously reported that S100A4 is expressed in both tumor cells, and endothelial cells [22, 23]. Although endothelial cells have not been shown to secrete S100A4, and its biological function in these cells is unknown, the endothelial S100A4 may be pertinent for the regulation of tumor angiogenesis and metastasis, and thus an attractive target for cancer therapy.

In this study, we used siRNA-mediated depletion and DNA microarray analysis to investigate the function of S100A4 in endothelial cells. Furthermore, the effect of S100A4 knockdown on angiogenesis and tumor growth was examined in a xenograft cancer model. The results suggest that S100A4 in endothelial cells is involved in tube formation and thus siRNA-mediated inhibition of endothelial S100A4 could provide an effective anti-tumor RNAi medicine.

## Materials and methods

### Cell culture

We cultured mouse endothelial cells (MSS31) [22, 24–26], human prostate carcinoma cells expressing a firefly luciferase gene (PC-3M-Luc C6, purchased from Caliper LifeSciences) [27], mouse melanoma cells (B16-BL6, whose origin and properties were described by Dr. Fidler [28], were a gift of Dr. S. Taniguchi, Shinsyu University Graduate School of Medicine) [29] and Lewis lung carcinoma cells (P29) [30] in DMEM supplemented with 10 % FBS.

### siRNA preparation

Synthetic 21-nt RNAs were purchased from Ambion/Life Technologies (Carlsbad, CA, USA). The murine S100A4 siRNA (mS100A4 siRNA) sequence was 5′-UGA ACA AGA CAG AGC UCA Att-3′ (sense) and 5′-UUG AGC UCU GUC UUG UUC Att-3′ (antisense). AllStars negative control siRNA (QIAGEN, Hilden, Germany) was used as a negative control.

### Transfection of siRNA in vitro

After 48 h of siRNA transfection using DharmaFECT No.1 (GE Healthcare, Waukesha, WI, USA), MSS31 cells were seeded on ECM-gel (SIGMA-Aldrich, St. Louis, MO, USA) thin-coated center-well dishes (60 mm, BD Falcon, Franklin Lakes, NJ, USA) at a cell density of 4 × 10^4^ cells/cm^2^ and cultured in the presence of 50 ng/ml HGF (PeproTech, Rocky Hill, NJ, USA). Capillary formation was assessed after 16 h of Matrigel culture [31]. RNA samples were extracted from transfected cells before seeding for real-time PCR analysis of S100A4 mRNA, using ISOGEN and platinum SYBR green qPCR superMix-UDG (Invitrogen/Life Technologies).

### Cell adhesion and wound healing assay

Migration ability was determined using a cell adhesion and a wound healing assay. After 48 h of S100A4 siRNA or negative control siRNA transfection, MSS31 cells was plated on 24-muti-well plate (Nunc, Thermo Scientific, Japan) at a cell density of 4 × 10^4^ cells/cm^2^ and then counted the number of adhered cells at 30 min, 1 and 3 h.

siRNA-transfected MSS31 cells were grown on 60 mm plates. After the cells reached sub-confluence, the cells were pretreated with mitomycin C (10 μg/ml) for 30 min and then the cells were wounded by scraping the monolayer and grown in medium for 16 h. The width of the wound was measured. Three different locations were visualized and photographed under a phase-contrast inverted microscope (ECLIPSE1000, Nikon, NY, USA). Data was represented as a percent of wound healing.

### Real-time PCR analysis

Total RNA was extracted from cells by ISOGEN (Nippon Gene, Tokyo, Japan) and treated with DNase I (Takara, Otsu, Japan). Five μg of total RNA was used to produce cDNAs with oligo (dT) 12 primer by superscript III RNA polymerase (Invitrogen/Life Technologies). cDNA was diluted five-fold and used for quantitative PCR. For quantitation, aliquots of 5 μl of cDNA samples were subjected to quantitative PCR in 50 μl reactions using Platinum Quantitative PCR SuperMix-UDG (Invitrogen/Life Technologies) and Assays-on-Demand TaqMan primers/probe sets (Applied Biosystems, Foster City, CA, USA) specific for target genes (Supplementary Table S1). Reactions were carried out using the ABI PRISM 7700 sequence detection System (Applied biosystems). The reactions were incubated at 50 °C for 2 min, then heated to 95 °C for 2 min followed by 45 cycles of 30 s at 95 °C, 15 s at 60 °C, and 20 s at 72 °C. The GAPDH gene and β-actin gene were used as an internal control.

### Microarray analysis

RNA quantity and quality were determined using a Nanodrop ND-1000 spectrophotometer (Thermo Fisher Scientific Inc., Waltham, MA, USA) and an Agilent Bioanalyzer (Agilent Technologies, Palo Alto, CA, USA), as per manufacturer’s instructions. Total RNA was amplified and labeled with Cyanine 3 (Cy3) using Agilent Low Input Quick Amp Labeling Kit, one-color (Agilent Technologies) according to the manufacturer’s instructions. Briefly, 100 ng of total RNA was reversed transcribed to double-strand cDNA, using a poly dT-T7 promoter primer. Primer, template RNA and quality-control transcripts of known concentration and quality were first denatured at 65 °C for 10 min, then incubated for 2 h at 40 °C with 5X first strand Buffer, 0.1 M DTT, 10 mM dNTP mix, and AffinityScript RNase Block Mix. The AffinityScript enzyme was then inactivated at 70 °C for 15 min. cDNA products were used as templates for in vitro transcription to generate fluorescent cRNA. cDNA products were mixed with a transcription master mix in the presence of T7 RNA polymerase and Cy3 labeled-CTP and incubated at 40 °C for 2 h. Labeled cRNAs were purified using QIAGEN’s RNeasy mini-spin columns and eluted in 30 μl of nuclease-free water. After amplification and labeling, cRNA quantity and cyanine incorporation were determined using a Nanodrop ND-1000 spectrophotometer and an Agilent Bioanalyzer. For each hybridization, 1.65 μg of Cy3 labeled cRNA was fragmented, and hybridized at 65 °C for 17 h on an Agilent Mouse GE 4x44K v2 Microarray (Design ID: 026655). After washing, microarrays were scanned using an Agilent DNA microarray scanner. Intensity values of each scanned feature were quantified using Agilent feature extraction software version 10.7.3.1, which performs background subtractions. Features that were flagged as having no errors (present flags) were used and features that were not positive, not significant, not uniform, or not above the background level were excluded. Features that were saturated and any population outliers (marginal and absent flags) were also excluded. Normalization was performed using Agilent GeneSpring GX version 11.0.2. (per chip: normalization to 75 percentile shift; per gene: normalization to median of all samples). There were 39,429 probes on the Agilent Mouse GE 4x44K v2 Microarray (Design ID: 026655), excluding control probes. The altered transcripts were quantified using the comparative method. A ≥ twofold change in signal intensity was designated as a significant difference in gene expression.

### Animals

Animal experiments were performed in compliance with the guidelines of the Institute for Laboratory Animal Research at the National Cancer Center Research Institute. The mice were maintained under specific pathogen-free conditions with a 12-h light–dark cycle.

### Study of xenografted tumors

Eight- to 11-week-old male athymic nude mice (CLEA Japan, Osaka, Japan) were used for all experiments. Anesthetized animals were subcutaneously injected with 3 × 10^6^ PC-3M-Luc C6 cells in 100 µl sterile Dulbecco’s PBS [30]. Nine days after subcutaneous inoculation of PC-3M-Luc C6 cells into mice (hereinafter referred to as day 1), tumors were treated twice with 50 μg siRNA/atelocollagen complex (day 1 and 3). After the treatment, tumor volume was measured at day 9, 12, and 15. Tumor size was measured with calipers and tumor volume was calculated by ab^2^/2, where a and b are the lengths of the long and short axes, respectively. Number of animals was 4 in each group and the experiment was performed twice.

### Imaging of tumor angiogenesis

Day 9 after the siRNA treatment, signals of AngioSense-IVM-750 were measured using fluorescence molecular tomography (FMT, VisEn Medical/PerkinElmer Inc., Waltham, MA, USA). AngioSense-IVM-750 is a 250 kDa macromolecule that freely circulates while staying confined to the intravascular space. Relative value of angiogenesis of mS100A4 siRNA-treated tumor was measured when the negative siRNA control was set to 1.0 and the data was represented as relative angiogenesis. Blood vessels in tumors monitored by Angiosence day 9 after the siRNA treatment, tumor vessels were also visualized using AngioSense-IVM-680 (ViSen medical) by intravenous administration of 50 μl per mouse. AngioSense-IVM-680 is a near-infrared labeled fluorescent macromolecule that remains localized in the vasculature for extended periods of time and enables imaging of blood vessels and angiogenesis. Fifteen min after the dye administration, the vessel images were acquired by using Olympus OV110 (Olympus, Japan).

### Atelocollagen-mediated siRNA delivery in vivo

To prepare the siRNA/atelocollagen complex, equal volumes of atelocollagen (1.0 % in PBS at pH 7.4, Koken Co., Ltd, Tokyo, Japan) and siRNA solution were combined and mixed by rotation for 20 min at 4 °C. The final concentration of atelocollagen was 0.5 %. Individual mice were injected with 200 μl of atelocollagen containing 50 μg of mS100A4 siRNA or non-silencing control siRNA/atelocollagen by intratumoral injection (negative control siRNA).

### Measurement of caspase-3/7 activity in vitro

Caspase-3/7, which plays key effector roles in apoptosis, was determined with the Apo-ONE Homogeneous caspase-3/7 assay (Promega) according to the manufacturer’s instructions. Cells were incubated with the Apo-ONE caspase-3/7 assay reagent for 1.5 h at room temperature, and the fluorescence was then measured at 485Ex/535Em with a Wallac multi-label counter.

### Double-immunostaining of CD31 and S100A4

B16-BL6 cells (1 × 10^6^ cells) were injected subcutaneously into C57BL/6 mice (6–7 weeks of age, Nippon SLC, Shizuoka, Japan). Fourteen days after the injection, tumor tissues were removed, embedded in a tissue-freezing medium, and then cut into 5 μm-thick sections. For double-immunostaining of CD31 and S100A4, tissue sections were fixed in acetone for 10 min. The samples were blocked with PBS containing 0.1 % bovine serum albumin and 2 % goat serum and then incubated with a mixture of rat anti-mouse CD31 antibody (Pharmingen, San Diego, CA, USA) and rabbit anti-S100A4 antibody [23]. The sections were washed three times with PBS, and incubated with a mixture of TRITC-labeled goat anti-mouse IgG and FITC-labeled goat anti-rabbit IgG. After rinsing with PBS, the sections were mounted in 50 % glycerol in PBS containing 1 mg/ml p-phenylenediamine to inhibit photobleaching, and were observed under a confocal laser scanning microscope (Fluoview, Olympus, Tokyo, Japan). Using NIH Image J 1.42q software (http://rsb.info.nih.gov/ij), a ratio of S100A4 pixel values to CD31 pixel values was calculated for each image (n = 6) for determination of S100A4/CD31 double-positive areas.

### Statistical analyses

Statistical analyses were conducted using Student’s *t* test for in vitro screening of cell capillary morphogenesis and proliferation and evaluation of in vivo angiogenesis. A *P* value of 0.05 or less was considered significant.

## Results

### Inhibition of capillary formation in endothelial cells by S100A4 siRNA

**Fig. 1:**
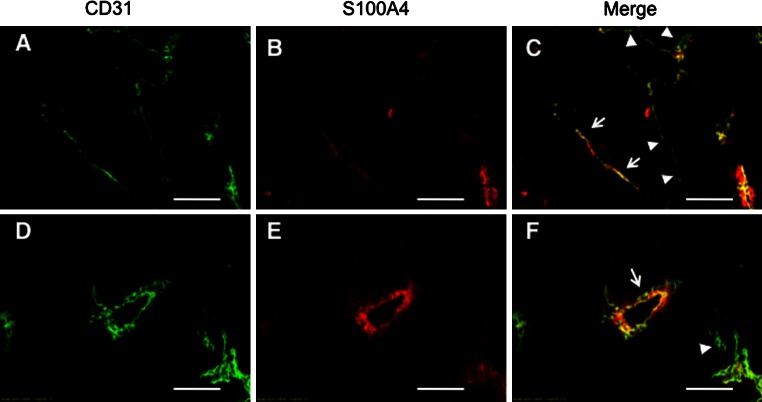
S100A4 expression in endothelial cells of intratumoral vessels. B16-BL6 cells were injected subcutaneously into C57BL/6 mice. Two frozen sections of the tumor tissue were double-immunostained with anti-CD31 antibody (**a**, **d**) and anti-S100A4 antibody (**b**, **e**). Merged imaged are also shown (**c**, **f**). *Arrows* and *arrowheads* in **c** and **f** indicate the examples of CD31^+^S100A4^+^ and CD31^+^S100A4^−^ microvessels, respectively. *Bars* 200 μm

**Fig. 2:**
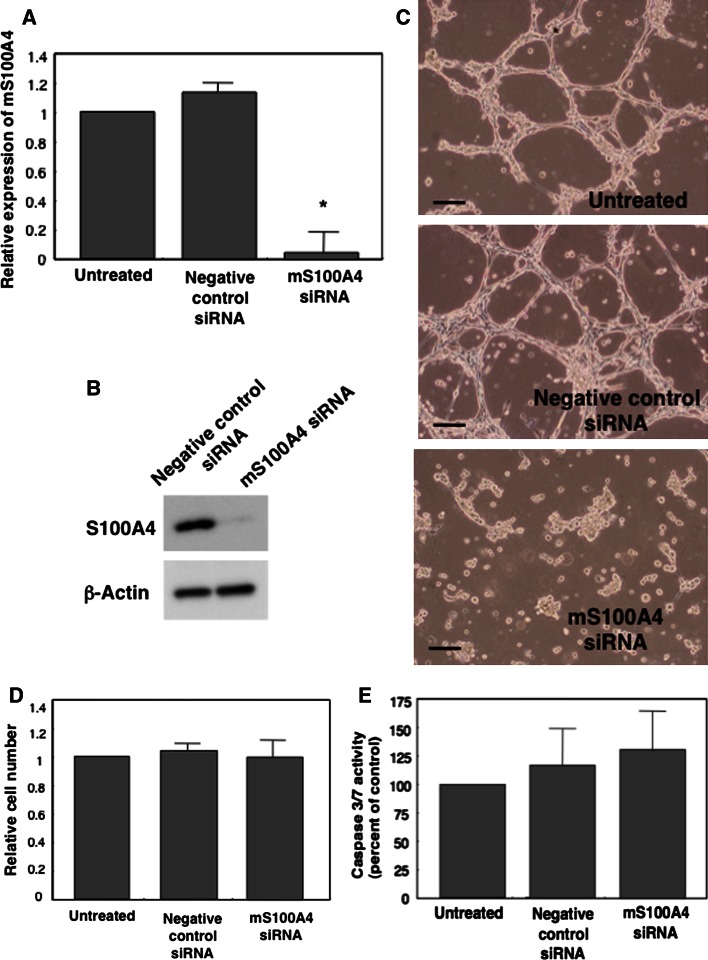
Anti-angiogenesis activity of S100A4 siRNA in vitro. MSS31 cells were transfected with murine S100A4 siRNA (mS100A4 siRNA) or control siRNA. Two days after transfection, 9.9 × 10^4^ MSS31 cells were seeded on ECM-gel thin-coated center-well dishes. RNA samples were extracted from transfected cells before seeding for real-time PCR analysis of S100A4 mRNA. HGF-induced capillary formation was assessed 16 h after Matrigel culture. **a** Relative expression of mS100A4 by real-time PCR analysis. *P = 0.01. **b** Western blot analysis of the expression of S100A4 in cells 24 h after siRNA transfection. Rabbit polyclonal anti-S100A4 antibody (Abcam, ab27957) and rabbit monoclonal anti-β actin antibody (Millipore, clone C4/MAB1501) were used. **c** Tube formation 16 h after siRNA treatment. Top, untreated control cells; middle, cells transfected with negative control siRNA; bottom, cells transfected with mS100A4 siRNA. Scale bars represent 50 μm. **d** Cell growth inhibition by mS100A4 siRNA. Cell growth was monitored by cell number analysis 16 h after siRNA transfection. **e** Caspase-3/7 activity. The activity was measured 16 h after siRNA transfection

**Fig. 3:**
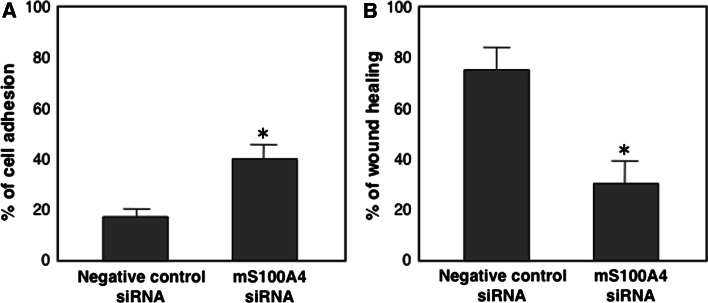
S100A4-mediated cell adhesion and migration. **a** Cell adhesion assay was performed on MSS31 cells treated with S100A4 siRNA or negative control siRNA (N.C. siRNA). Phase-contrast photograph of adhered cells was recorded at 30 min, 1 and 3 h after cell plating. Percent of cell adhesion at 30 min was exhibited. S100A4 siRNA-treated cells: 41 ± 5 %; negative control siRNA-treated cells: 18 ± 2 %, *P* = 0.0017. **b** Wound healing assay was performed on MSS31 cells treated with S100A4 siRNA or N.C. siRNA. The width of the wound was measured after 16 h of the cell wounding by scraping the monolayer. Three different locations were visualized and photographed under a phase-contrast inverted microscope (ECLIPSE1000, Nikon). Data was represented as a percent of wound healing. S100A4 siRNA-treated cells: 30 ± 7 %; negative control siRNA-treated cells: 76 ± 9 %, *P* = 0.0003

We first examined whether endothelial cells of tumor microvessels express S100A4. For this, we immunostained the microvessels in tumor tissues formed by B16-BL6 melanoma cells that express little S100A4 with anti-CD31 and anti-S100A4 antibody (Fig. 1, Supplementary Fig. S1). The results showed that there were S100A4-positive and -negative CD31^+^ endothelial cells (arrows and arrowheads in Fig. 1, panels c and f). Quantification of each S100A4^+^ and CD31^+^ area in double-stained tissue sections showed that approximately half (49.3 ± 29.5 %, n = 6) of CD31^+^ endothelial cells was S100A4-positive. These results suggest that there exist subpopulations of endothelial cells in tumors that might, or might not, be primed for angiogenesis. This prompted us to examine the role of S100A4 in angiogenesis and, to this end, we tested the effect of siRNA-mediated depletion of S100A4 on capillary formation in mouse endothelial MSS31 cells. Specifically, murine S100A4 siRNA (mS100A4 siRNA) completely blocked S100A4 expression in MSS31 cells at both the mRNA and protein levels (Fig. 2a, b). Hepatocyte growth factor (HGF)-induced capillary formation was assessed 16 h after Matrigel culture [2]. siRNA-induced knockdown of mS100A4 resulted in the inhibition of HGF-induced capillary formation in MSS31 cells in vitro, while control siRNA showed no inhibitory effect when compared to untreated controls (Fig. 2c). Additionally, suppression of cell growth of MSS31 cells was not detectable within 16 h of mS100A4 siRNA treatment (Fig. 2d) and the analysis of caspase 3/7 activity did not show caspase-dependent apoptotic cell death (Fig. 2e), excluding a possibility that the inhibition of tube formation by the siRNA is non-specific effect. These results indicate that S100A4 is important for tube formation of endothelial cells. In addition, cell adhesion and cell migration assay was performed. As shown in Fig. 3a, cell adhesion was significantly enhanced by inhibition of S100A4 by S100A4 siRNA as compared to N.C. siRNA (*P* = 0.0017). Wound healing assay revealed that knockdown of S100A4 inhibited cell migration significantly (*P* = 0.0003) in Fig. 3b. Taken together, these results of multiple angiogenesis assay clearly indicate that S100A4 is significantly involved in angiogenesis.

### Inhibition of tumor angiogenesis by S100A4 siRNA

**Fig. 4:**
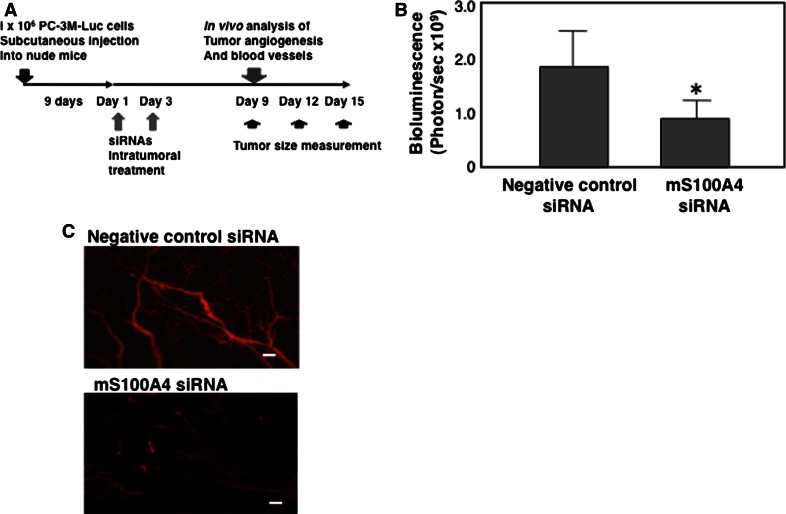
Inhibition of tumor angiogenesis by mouse S100A4-specific siRNA (mS100A4 siRNA). **a** Schematic representation of in vivo animals experiments. PC-3M-Luc C6 cells (1 × 10^6^ cells) were subcutaneously inoculated into 9-week-old male athymic nude mice. Nine days after subcutaneous inoculation of PC-3M-Luc C6 cells into mice (hereinafter referred to as day 1), tumors were treated twice with 50 μg siRNA/atelocollagen complex (day 1 and 3). Number of animals in each group was 4. **b** At day 9, angiogenesis in tumors was monitored with the administration of the fluorescence detection agent, AngioSence-IVM-750, by FMT. Photon count per sec of mS100A4 siRNA-treated and the negative control siRNA-treated tumors were exhibited. The animal experiment was performed twice (N = 4 in each experiment). *P = 0.05. **c** At day 9, angiogenesis in tumors monitored by Angiosence. Vessels were visualized using AngioSense-IVM-680 (ViSen medical) by intravenous administration of 50 μl per mouse. Fifteen min after the dye administration, the vessel images were acquired by using Olympus OV110 (Olympus, Japan). Scale bars represent 100 μm

**Fig. 5:**
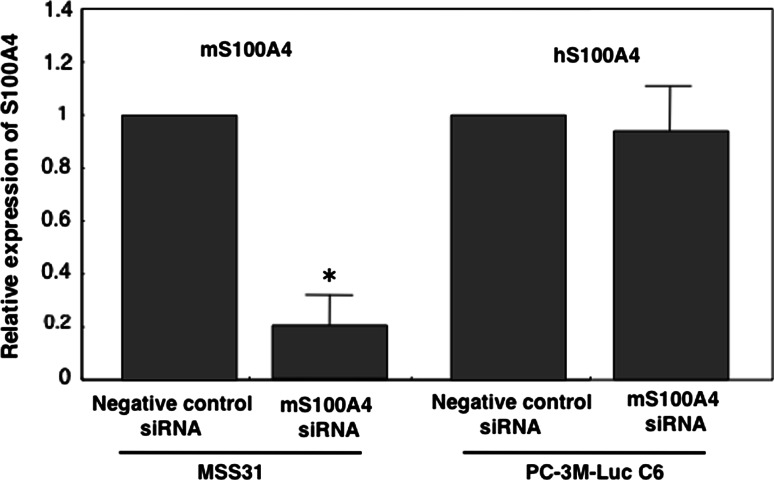
mS100A4 siRNA specifically inhibited expression of mS100A4, but not human S100A4 (hS100A4). MSS31 (murine) and PC-3M-Luc C6 (human) cells were transfected with murine S100A4 siRNA (mS100A4 siRNA) and negative control siRNA. Two days after transfection, RNA samples were extracted from transfected cells and subjected to real-time PCR analysis of S100A4 mRNA. *P = 0.05

To examine the effect of mS100A4 siRNA on tumor angiogenesis, PC-3M-Luc C6 cells were inoculated into athymic mice. Nine days after the injection (hereinafter referred to as day 1), tumors were treated twice with 50 μg siRNA/atelocollagen complex intratumorally. After the treatment, tumor angiogenesis was measured at day 9 (Fig. 4a). Administration of a fluorescent imaging agent revealed a strong inhibition of tumor angiogenesis by mS100A4 siRNA (Fig. 4b), without a discernible reduction in tumor volume (Supplementary Fig. S3). The mS100A4 siRNA was designed to inhibit specifically murine S100A4 expression, and not human S100A4 in PC-3M-Luc C6 cells (Fig. 5). Thus, these findings showed that endothelial cells were the primary targets of mS100A4 siRNA, not human tumor cells. Additionally, AngioSense analysis demonstrated that vessel formation in tumors was clearly inhibited by mS100A4 siRNA (Fig. 4c, Supplementary Fig. S2, Table 1). Thus, the results indicate that mS100A4 siRNA acts directly upon and inhibits host angiogenesis. We also found that mS100A4 siRNA inhibits tumor growth, with a significant reduction in tumor volume by day 15 (Table 2).

**Table 1:** Area of vessels in animals

	Volume of tumor (mm^3^)	Area of vessels (mm^2^)
Negative control siRNA	36.3 ± 3.1	0.129 ± 0.001
mS100A4 siRNA	31.5 ± 5.0	0.053 ± 0.0008*

Nine days after subcutaneous inoculation of human prostate cancer cells (PC-3M-Luc C6) into mice (hereinafter referred to as day 1), tumors were treated twice with 50 µg siRNA/atelocollagen complex. After the treatment angiogenesis images were acquired at day 9 using Olympus OV110 and vessels were visualized using AngioSense-IVM-680 (ViSen medical) by intravenous administration. Area of vessels was measured by Image J (Olympus). Number of animals was 4 in each group

* *P* < 0.05

**Table 2:** Tumor volume in animals

Volume of tumor (mm^3^)	Day 9	Day 12	Day 15
Negative control siRNA	36.3 ± 3.1	39.7 ± 2.6	44.8 ± 1.8
mS100A4 siRNA	31.5 ± 5.0	30.2 ± 3.8	27.1 ± 2.2*

Nine days after subcutaneous inoculation of human prostate cancer cells (PC-3M-Luc C6) into mice (hereinafter referred to as day 1), tumors were treated twice with 50 µg siRNA/atelocollagen complex twice (day 1 and 3). After the treatment tumor volume was measured at day 9, 12, and 15. Number of animals was 4 in each group

* *P* < 0.05

### Microarray analysis of angiogenesis-related gene expression in S100A4 knockdown endothelial cells

**Fig. 6:**
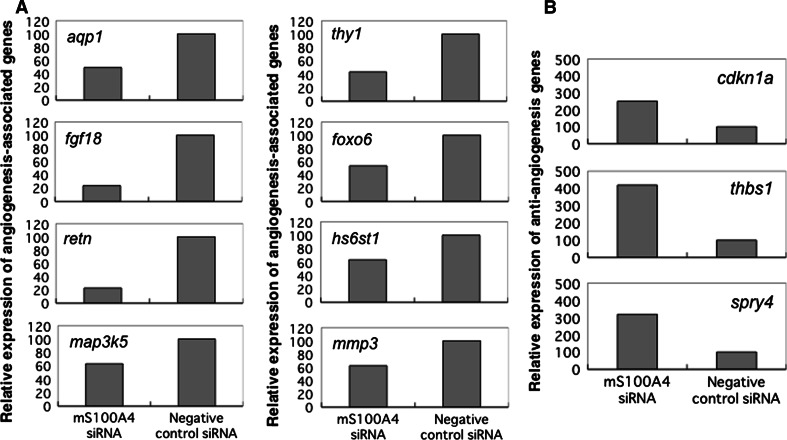
Modulation of the expression of angiogenesis-related genes by S100A4 siRNA. Verification of microarray analysis via RT-PCR. RT-PCR analysis was performed under linear amplification conditions. The *GAPDH* gene was used as an internal control. **a** Genes in mS100A4 knockdown cells with lower expression levels included angiogenesis-associated genes. **b** Genes in mS100A4 knockdown cells with higher expression levels included anti-angiogenesis genes

To gain mechanistic insight into the above observations, we compared the gene expression profiles of MSS31 cells treated with either mS100A4 siRNA or control siRNA. Unsupervised hierarchical cluster analysis was performed on 745 angiogenesis-related genes (Supplementary Fig S4,Supplementary Table S1, Supplementary excel file). The analyses showed that significant alterations in angiogenesis-related gene expression occurred in the S100A4 siRNA treatment group, suggesting that S100A4 plays a role in controlling angiogenesis. Genes with significant alterations (twofold or more) in expression level are listed in Table 3. These genes were validated by qRT-PCR analysis (Fig. 6a, b). Genes in mS100A4 knockdown cells with lower expression levels included angiogenesis-associated genes, such as aquaporin-1 (*aqp1*) [32], fibroblast growth factor 18 (*fgf18*) [33], resistin (*retn*) [34], mitogen-activated protein kinase kinase kinase 5 (*map3k5*) [35], thymus cell antigen (*thy1*) [36], forkhead box O6 (*foxo6*) [37], heparan sulfate 6-O-sulfotransferase 1 (*hs6st1*) [38] and matrix metalloproteinase 3 (*mmp3*) [39], which are widely expressed in cardiovascular tissue. They induce endothelial migration and microvessel formation, which are central to diverse biological phenomena including angiogenesis, wound healing, and metastasis. Genes in mS100A4 knockdown cells with higher expression levels included anti-angiogenesis genes such as cyclin-dependent kinase inhibitor 1A (*cdkn1a*) [40], thrombospondin 1 (*thbs1*) [41], and sprouty homolog 4 (*spry4*) [42]. Taken together, microarray analysis validated the function of endothelial S1004A in angiogenesis.

**Table 3:** Genes of significant two fold or more alteration of the expression level in mS100A4 knockdown endothelial MSS31 cells

Gene name	Gene symbol	Fold change
Downregulated gene		
Resistin	retn	4.8
Fibroblast growth factor 18	fgf18	4.6
Aquaporin-1	aqp1	2.9
Mitogen-activated protein kinase kinase kinase 5	map3k5	2.2
Thymus cell antigen	thy1	2.1
Forkhead box 06	foxo6	2.1
Heparan sulfate 6-O-sulfotranseferase 1	hs6st1	2.0
Matrix metalloproteinase 3	mmp3	1.7
Upregulated gene		
Cyclin-dependent kinase inhibitor 1A	cdkn1a	3.2
Thrombospondin 1	thbs1	2.4
Sprouty homolog 4	spry4	2.4

## Discussion

Using B16BL6 tumor tissues little expressing S100A4, we stained tumor microvessels for CD31 and S100A4 and found that there are subpopulations of endothelial cells in tumors, S100A4-positive and –negative ones. This observation motivated us to examine a possible role of endothelial S100A4 by silencing it. The multiple angiogenesis assay including tube formation, adhesion, and migration analysis of endothelial cells clearly indicated that endothelial S100A4 plays a crucial role in angiogenesis. S100A4-positive endothelial cells in tumors may represent the ones primed for neoangiogenesis. A comparison of the gene expression profiles of siRNA-treated cells with those of untreated cells showed that endothelial S100A4 acts upstream of a variety of angiogenesis-related genes. These findings were confirmed in a xenograft tumor model, where intratumor administration of siRNA distinctly reduced tumor angiogenesis and growth.

In the present study, mouse siRNA was delivered in vivo using atelocollagen, a highly purified type I collagen with low immunogenicity. Atelocollagen forms nano-sized particles when mixed with oligonucleotides such as double stranded RNAs and DNAs via electrostatic binding, and is incorporated into cells by endocytosis [43, 44]. In xenografted tumor tissues, many cell types can take up the complex, including human prostate cancer cells, endothelial cells and stromal cells. However, the specificity of the siRNA for mouse S100A4 suggests that the primary target of the S100A4 siRNA was the mouse vasculature.

Microarray analysis further confirmed the molecular mechanism of S100A4-mediated angiogenesis in endothelial cells. Significant changes in angiogenesis-promoting gene expression occurred in S100A4 siRNA-treated endothelial cells. Among the genes exhibiting altered expression levels, *aqp1*, *map3k5*, and *fgf18* are highly expressed in tumor-associated blood vessels in several human tumors [45–48]. Furthermore, our results indicate that S100A4 may negatively regulate anti-angiogenic genes, such as *cdkn1a*, *thbs1*, and *spry4*. This means that S100A4 has a dual effect on angiogenesis-related gene expression: it up-regulates angiogenic genes and down-regulates anti-angiogenic ones, thereby determining the fate of endothelial cells in association with tumorigenesis. The biological significance of all these genes has been proven in vitro and in vivo analysis of angiogenesis in previous reports.

Our sequential observations revealed that mS100A4 knockdown induced apparent cell growth inhibition of MSS31 cells in a later stage after siRNA treatment. It suggests a possible role of S100A4 in regulating endothelial cell growth, which may, in part, account for a drastic inhibition of neoangiogenesis in mS100A4 siRNA-administered tumor. Further analysis is required to know whether S100A4 promotes growth and survival of neovascular endothelial cells.

In the human cancer xenograft model used in this study, only host S100A4 was targeted to explore the role of S100A4 in angiogenesis. However, S100A4 in tumor cells is also involved in invasion and metastasis. Therefore, it would be interesting to investigate whether administration of an agent that inhibits both mouse and human S100A4 could suppress the metastasis of xenografted human tumor cells in immuno-compromised mice. If suppression occurs, it would suggest that inhibition of S100A4 could reduce tumor angiogenesis and by inference, tumor metastatic ability, which would have important implications for the development of cancer therapeutics. Furthermore, it would underscore the advantages of S100A4 inhibition over conventional angiogenesis factor/receptor inhibition. Studies are currently under way to develop such agents.

In conclusion, the present results show the importance of endothelial S100A4 expression in tumor angiogenesis. As the generation of a new vascular supply is causally involved in the progression of the majority of solid tumors, siRNA-mediated inhibition of endothelial S100A4 could provide an effective anti-tumor RNAi medicine. Thus, the development of inhibitors targeting endothelial S100A4 could represent a promising approach for effective anti-angiogenesis and anti-metastasis therapy.

## Electronic supplementary material

Below is the link to the electronic supplementary material.

RT-PCR analysis of the expression of S100A4 in B16-BL6 melanoma cells. Total RNA isolated from Lewis lung carcinoma P29 cells and B16-BL6 melanoma cells was subjected to RT-PCR analysis. *GAPDH* and *β*-*actin* were used as quality and loading controls. P29 cells were used as a positive control for S100A4 expression [26]. B16-BL6 cells expressed little S100A4 mRNA. In accordance with this result, S100A4 was hardly detected in B16-BL6 tumor sections by immunohistochemistry as shown in Fig. 1 (TIFF 1521 kb)

Relative angiogenesis was measured by signals of AngioSense-IVM-750 using FMT. Relative value of angiogenesis of mS100A4 siRNA-treated tumor when the negative siRNA control was set to 1.0. *P = 0.05. Number of animals (11-week-old male athymic nude mice) in each group was 4 (TIFF 1521 kb)

Short-term effect of mS100A4 siRNA on the growth of xenografted human tumor. Atelocollagen/mS100A4 siRNA or negative control siRNA complex was delivered into the xenografted human tumor, and the tumor volume was assessed 2 days after the siRNA administration. Relative tumor volume of mice 2 days after siRNA treatment is shown. An apparent reduction of tumor volume on day 2 after the mS100A4-knockdown was not evident (TIFF 1521 kb)

Unsupervised hierarchical analysis of 745 gene expression profiles and candidate validation. Data were subjected to a hierarchical cluster analysis using a Euclidean distance calculation based on the unweighted pair group method with arithmetic mean. The samples were aligned horizontally: lane S, mS100A4 siRNA-treated; lane A, negative control siRNA-treated; lane R, transfection reagent alone; and lane N, negative control siRNA alone. Red, yellow, and blue color areas represent high, middle, and low expression levels, respectively (TIFF 1521 kb)

The gene list from 745 angiogenesis-related genes used for unsupervised hierarchical cluster analysis (TIFF 1521 kb)
