# Supplementary figures and images for: Silencing of S100A4, a metastasis-associated protein, in endothelial cells inhibits tumor angiogenesis and growth
Source: Angiogenesis. 2013 Aug 9;17(1):17–26. doi: 10.1007/s10456-013-9372-7 (PMC3898373; doi:10.1007/s10456-013-9372-7)

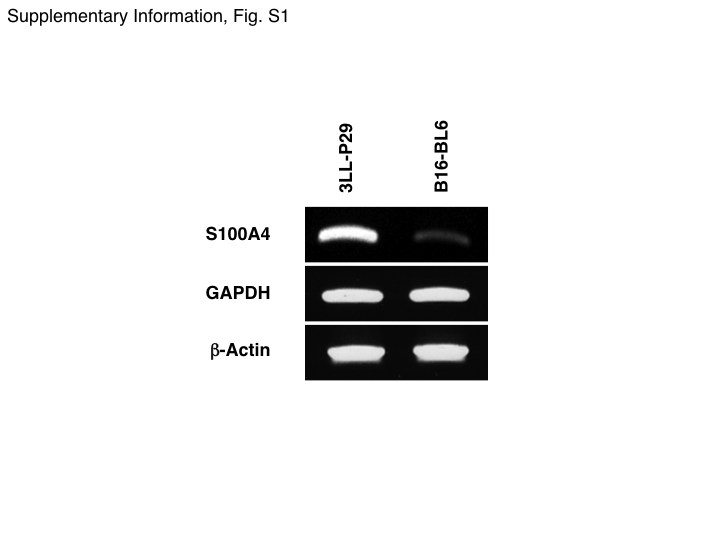

Supplement: Supplementary file 1 — RT-PCR analysis of the expression of S100A4 in B16-BL6 melanoma cells. Total RNA isolated from Lewis lung carcinoma P29 cells and B16-BL6 melanoma cells was subjected to RT-PCR analysis. GAPDH and β-actin were used as quality and loading controls. P29 cells were used as a positive control for S100A4 expression [26]. B16-BL6 cells expressed little S100A4 mRNA. In accordance with this result, S100A4 was hardly detected in B16-BL6 tumor sections by immunohistochemistry as shown in Fig. 1 (TIFF 1521 kb) [file 10456_2013_9372_MOESM1_ESM.tif]

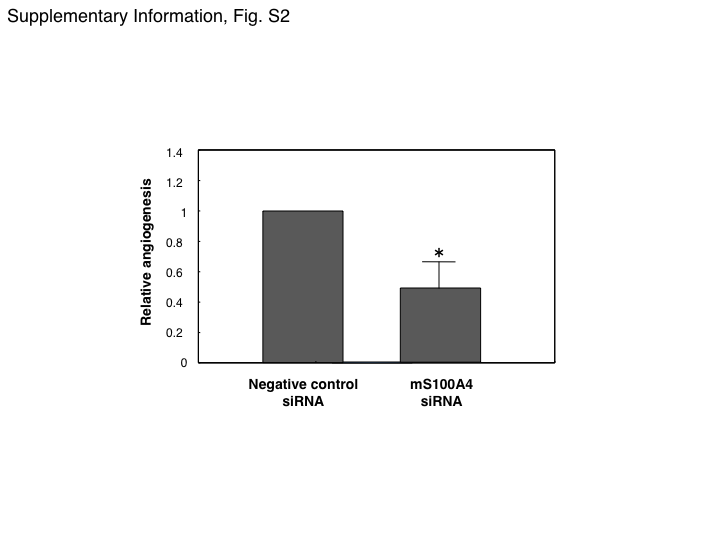

Supplement: Supplementary file 2 — Relative angiogenesis was measured by signals of AngioSense-IVM-750 using FMT. Relative value of angiogenesis of mS100A4 siRNA-treated tumor when the negative siRNA control was set to 1.0. *P = 0.05. Number of animals (11-week-old male athymic nude mice) in each group was 4 (TIFF 1521 kb) [file 10456_2013_9372_MOESM2_ESM.tif]

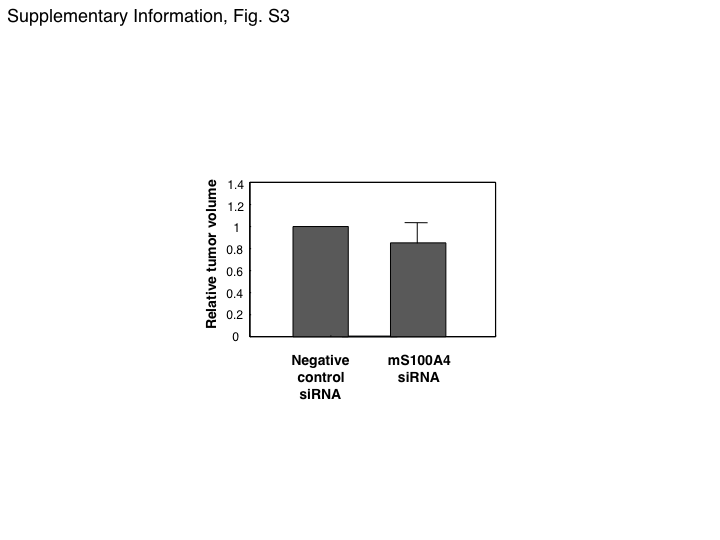

Supplement: Supplementary file 3 — Short-term effect of mS100A4 siRNA on the growth of xenografted human tumor. Atelocollagen/mS100A4 siRNA or negative control siRNA complex was delivered into the xenografted human tumor, and the tumor volume was assessed 2 days after the siRNA administration. Relative tumor volume of mice 2 days after siRNA treatment is shown. An apparent reduction of tumor volume on day 2 after the mS100A4-knockdown was not evident (TIFF 1521 kb) [file 10456_2013_9372_MOESM3_ESM.tif]

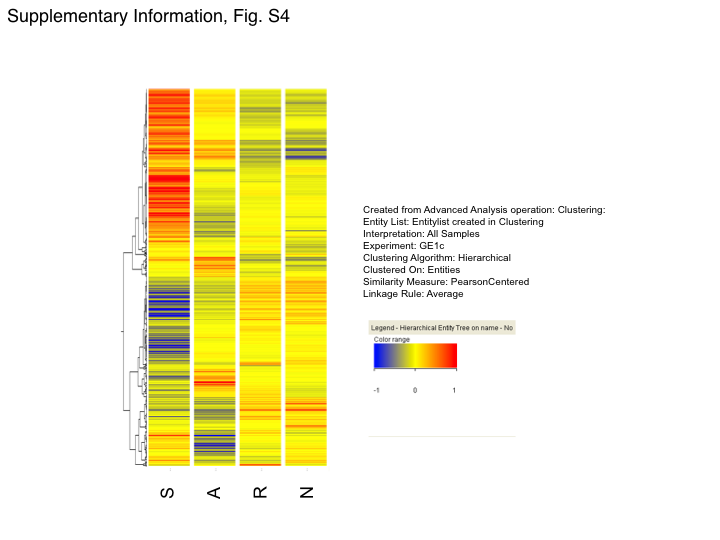

Supplement: Supplementary file 4 — Unsupervised hierarchical analysis of 745 gene expression profiles and candidate validation. Data were subjected to a hierarchical cluster analysis using a Euclidean distance calculation based on the unweighted pair group method with arithmetic mean. The samples were aligned horizontally: lane S, mS100A4 siRNA-treated; lane A, negative control siRNA-treated; lane R, transfection reagent alone; and lane N, negative control siRNA alone. Red, yellow, and blue color areas represent high, middle, and low expression levels, respectively (TIFF 1521 kb) [file 10456_2013_9372_MOESM4_ESM.tif]

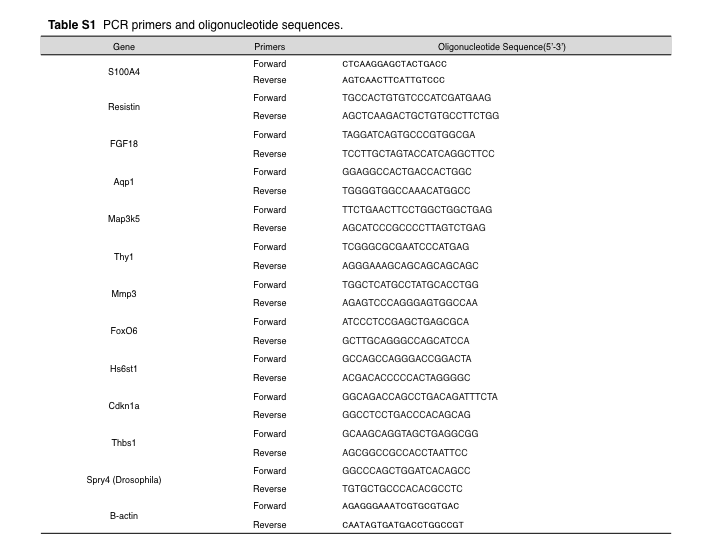

Supplement: Supplementary file 5 — The gene list from 745 angiogenesis-related genes used for unsupervised hierarchical cluster analysis (TIFF 1521 kb) [file 10456_2013_9372_MOESM5_ESM.tif]
